# m^6^A RNA Methylation in Psychiatric Disorders: An Emerging Epitranscriptomic Axis

**DOI:** 10.3390/epigenomes9030036

**Published:** 2025-09-19

**Authors:** Ambrose Loc Ngo, Linda Nguyen, Niki Gharavi Alkhansari, Huiping Zhang

**Affiliations:** 1 College of Osteopathic Medicine, Kansas City University, Farber-McIntire Campus, 2901 St. Johns Blvd, Joplin, MO 64804, USA; ambroseloc.ngo@kansascity.edu (A.L.N.); niki.gharavi@kansascity.edu (N.G.A.); 2 College of Pharmacy, Western University, California Campus, 309 E. Second St., Pomona, CA 91766-1854, USA; linda.nguyen2@westernu.edu; 3 Department of Psychiatry, Boston University Chobanian & Avedisian School of Medicine, 72 East Concord Street, Boston, MA 02118-2526, USA; 4 The Biomedical Genetics Section, Department of Medicine, Boston University Chobanian & Avedisian School of Medicine, 72 East Concord Street, Boston, MA 02118-2526, USA

**Keywords:** m^6^A RNA methylation, epitranscriptomics, gene expression regulation, neurodevelopment, psychiatric disorders

## Abstract

N^6^-methyladenosine (m^6^A) is the most prevalent internal modification in eukaryotic messenger RNA (mRNA) and plays a vital role in post-transcriptional gene regulation. In recent years, m^6^A has emerged as a pivotal epitranscriptomic signal involved in neural development, synaptic remodeling, and the molecular pathophysiology of neuropsychiatric disorders. In this review, we summarize the mechanisms underlying the deposition, removal, and recognition of m^6^A by dedicated methyltransferases, demethylases, and RNA-binding proteins. We further explore how these dynamic modifications influence neuronal differentiation and memory formation. Recent studies have linked aberrant m^6^A regulation to psychiatric conditions such as depression, anxiety, schizophrenia, and bipolar disorder. Additionally, we discuss how pharmacological or genetic modulation of m^6^A pathways may promote adaptive neural plasticity and enhance cognitive and emotional resilience. Despite these promising findings, significant challenges remain in achieving spatial and temporal specificity while minimizing off-target effects in the brain. Therefore, we advocate for more in-depth investigations into m^6^A function within developmentally defined neural circuits to better understand its enduring role in maintaining neural homeostasis.

## 1. Introduction

RNA modifications are redefining the landscape of gene regulation. At the forefront of this epitranscriptomic revolution is N^6^-methyladenosine (m^6^A), the most abundant internal modification in eukaryotic messenger RNA (mRNA) [1,2]. m^6^A is estimated to occur in approximately 0.1–0.4% of all adenosines in mammalian mRNAs, typically amounting to ~3–5 m^6^A sites per transcript, with enrichment near stop codons, in 3′ UTRs, and in long internal exons [3]. The dynamic installation and removal of m^6^A are orchestrated by a tripartite enzymatic network: methyltransferase dimers, primarily METTL3 (methyltransferase-like 3) and METTL14 (methyltransferase-like 14), serve as “writers”; the demethylases FTO (fat mass and obesity-associated protein) and ALKBH5 (alkB homolog 5) act as “erasers”; and “reader” proteins, notably those containing YTH domains, interpret the methyl mark to direct downstream outcomes [4]. This reversible chemical code modifies RNA without altering its sequence, adding a regulatory layer that is both flexible and tightly controlled [4]. m^6^A influences virtually every stage of mRNA metabolism, making its biological impact far-reaching. In the central nervous system (CNS), m^6^A is not merely an accessory mechanism but a key determinant of transcript behavior [5,6]. Genetic and pharmacological studies have shown that disruption of m^6^A pathways impairs embryonic neurogenesis, interferes with synaptic plasticity underlying learning, and compromises the adaptive remodeling of neural circuits [5,6,7]. These findings suggest that m^6^A represents a molecular “grammar” by which chemical modifications are translated into adaptive, behaviorally relevant neural responses.

Altered expression or activity of m^6^A-modifying enzymes has been linked to dysregulated neural gene expression, aberrant synaptic connectivity, and disrupted stress response circuitry [5,8]. These discoveries position m^6^A as a critical molecular nexus linking genetically encoded programs with dynamic environmental signals in the CNS [9]. Nonetheless, the precise mechanisms through which m^6^A influences the onset and progression of neuropsychiatric disorders remain incompletely understood. A major challenge lies in elucidating the cell-type-specific and circuit-level consequences of m^6^A signaling in the brain’s complex cellular environment. Moreover, understanding how environmental perturbations, such as chronic stress or repeated drug exposure, intersect with m^6^A dynamics may reveal novel insights into neural plasticity and vulnerability to mental illness.

This review seeks to bridge molecular mechanisms with clinically observable outcomes. We summarize recent advances clarifying m^6^A’s role in neuronal differentiation, synaptic plasticity, memory stabilization, and reward circuitry, and we explore how m^6^A dysregulation contributes to psychiatric disorders. Finally, we discuss the emerging potential of m^6^A-modifying enzymes and reader proteins as biomarkers and therapeutic targets in psychiatry and neurology, positioning the m^6^A epitranscriptome at the nexus of basic research and clinical translation.

## 2. Mechanisms of RNA Methylation

### 2.1. Overview of m^6^A Machinery

RNA methylation plays crucial roles in regulating cellular processes such as proliferation, differentiation, and stress responses [10]. It is mediated by a dynamic network of proteins commonly categorized as “writers,” “erasers,” and “readers”. Methyltransferases (“writers”) and demethylases (“erasers”) regulate the reversible methylation of N^6^-methyladenosine (m^6^A), the most abundant internal modification in eukaryotic mRNA [11]. Transcriptome-wide mapping of m^6^A has shown that these sites are significantly enriched in certain 5′-DRA*CH-3′ sequences (where A* denotes the methylatable adenosine; D represents A, G, or U; R represents A or G; and H represents A, C, or U) [12,13]. The m^6^A methyltransferase complex (MTC) comprises three core catalytic subunits [METTL3 (methyltransferase-like 3), METTL14 (methyltransferase-like 14), and WTAP (Wilms tumor 1-associating protein)] alongside four regulatory subunits [VIRMA (also known as KIAA1429 or Vir-like m^6^A methyltransferase associated), ZC3H13 (zinc finger CCCH-type containing 13), HAKAI (also known as Cbl proto-oncogene like 1 or CBLL1), and RBM15 (RNA binding motif protein 15)] [11]. The core MTC, composed of METTL3, METTL14, and WTAP, catalyzes the methylation of adenosine residues in RNA. Regulatory proteins such as VIRMA, ZC3H13, HAKAI, and RBM15 enhance the specificity and subcellular localization of the complex. METTL3 functions as the primary catalytic component, METTL14 stabilizes METTL3 and enhances RNA binding, and WTAP acts as a scaffold protein critical for nuclear localization of the complex [14]. The regulatory subunits modulate the complex’s specificity and subcellular targeting.

Transcriptomic analyses using PAR-CLIP (photoactivatable ribonucleoside-enhanced crosslinking and immunoprecipitation) have shown that WTAP and METTL3 cooperatively regulate gene expression and alternative splicing, especially for genes involved in transcription and RNA processing [15]. WTAP forms a stable complex with METTL3 and METTL14 and is essential for localizing the MTC to nuclear speckles, subnuclear domains enriched in pre-mRNA processing factors. WTAP is also required for the catalytic activity of METTL3; its absence significantly reduces METTL3’s RNA-binding capacity, suggesting it is essential for guiding the MTC to its mRNA targets [15].

“Reader” proteins, particularly those in the YTH domain family, recognize m^6^A-modified RNA and mediate its downstream effects. These include YTHDF1, YTHDF2, YTHDF3 (YTH N^6^-methyladenosine RNA binding proteins F1-F3), as well as YTHDC1 and YTHDC2 (YTH domain-containing proteins C1 and C2). YTH proteins bind m^6^A-modified RNA via a conserved YTH domain containing a hydrophobic binding pocket, often referred to as an “aromatic cage” [11]. This cage, formed by conserved aromatic residues, enables selective recognition of methylated RNA through π–π stacking and hydrogen bonding, distinguishing it from unmethylated transcripts [10]. The YTHDF proteins carry out distinct cellular functions due to differences in their low-complexity N-terminal regions: YTHDF1 primarily enhances translation, YTHDF2 promotes mRNA degradation, and YTHDF3 acts as a modulator, cooperating with both YTHDF1 and YTHDF2 to coordinate the translation and decay of target mRNAs [16,17]. YTHDC1 functions mainly in the nucleus, where it regulates mRNA splicing, export, and nuclear retention, whereas YTHDC2 operates in both the nucleus and cytoplasm to regulate mRNA stability and translation. However, its m^6^A-binding capacity is less well established, and it also possesses RNA helicase activity [18,19]. Additional m^6^A reader proteins include the insulin-like growth factor-2 mRNA-binding proteins 1, 2, and 3 (IGF2BP1, IGF2BP2, IGF2BP3), as well as heterogeneous nuclear ribonucleoproteins (HNRNPs) such as HNRNPA2B1 and HNRNPC. Members of the IGF2BP family bind m^6^A-modified RNA and promote mRNA stability and translation [20,21], whereas nuclear readers HNRNPA2B1 and HNRNPC recognize m^6^A-containing sites on nascent transcripts to regulate alternative splicing [22,23]. Together, these m6A reader proteins shape RNA fate and gene expression, thereby influencing diverse biological processes, including development, metabolism, stress responses, and disease.

m^6^A is a reversible mark, removed by demethylases or “erasers” such as fat mass and obesity-associated protein (FTO) and alkB homolog 5 (ALKBH5) [24]. FTO, the first identified m^6^A demethylase, catalyzes oxidative demethylation of m^6^A, affecting RNA stability and splicing [24]. Overexpression of FTO reduces global m^6^A levels, while its downregulation leads to increased m^6^A methylation [25]. Li et al. demonstrated that FTO depletion increases m^6^A modifications on mRNAs involved in ribosome biogenesis, thereby promoting their YTHDF2-mediated decay [26]. Similarly, ALKBH5 removes m^6^A marks and modulates nuclear RNA export, mRNA metabolism, and gene expression [27].

### 2.2. RNA Methylation in mRNA Regulation

Advances in epitranscriptomics have underscored the importance of post-transcriptional modifications in shaping gene expression in the nervous system. Chemical modifications such as m^6^A, N^1^-methyladenosine (m^1^A), and 5-methylcytosine (m^5^C), dynamically influence mRNA stability, translation, splicing, and intracellular localization. These reversible marks add a critical layer of control to mRNA function, governing key processes like neuronal development, synaptic plasticity, and the stress response.

### 2.3. m^6^A: A Central Epitranscriptomic Mark in the Nervous System

Among all known mRNA modifications, m^6^A is the most prevalent and well-characterized internal mark. It regulates transcript fate at multiple levels, including splicing, stability, transport, and translation, making it essential for neuronal function [28]. Emerging evidence suggests that m^6^A also plays a role in maintaining neural cell identity and differentiation [29]. m^6^A methylation affects the stability, splicing, translation, and subcellular localization of mRNA. In neurons, m^6^A-marked transcripts tend to localize to neurites, implicating this modification in local protein synthesis at synapses—crucial for synaptic plasticity and signaling [30]. FTO, by removing m^6^A near splice sites, modulates alternative splicing outcomes by influencing spliceosome assembly [25]. m^6^A also regulates alternative splicing through interactions with splicing factors like SRSF2 and influences translation efficiency by recruiting initiation factors. This regulation is particularly important in neurons, where rapid and spatially restricted protein synthesis is necessary for functional adaptation. In specific contexts, such as when m^6^A sites are located within coding regions, this modification can trigger translation-dependent mRNA decay, adding a level of fine-tuned control over transcript lifespan [29]. Additionally, m^6^A RNA methylation, particularly mediated by METTL3, promotes neuroinflammation by enhancing activation of the TRAF6–NF-κB pathway [31,32]. Through increasing the stability and/or translation of TRAF6 mRNA, m^6^A leads to elevated TRAF6 protein expression. The upregulated TRAF6 then activates NF-κB signaling, which drives the production of pro-inflammatory cytokines and thereby exacerbates neuroinflammatory responses.

### 2.4. m^1^A: A Marker of Neuronal Stress Adaptation

N^1^-methyladenosine (m^1^A) is another important epitranscriptomic mark, particularly in neuronal responses to stress [33]. In models of oxygen-glucose deprivation and reoxygenation (OGD/R), m^1^A levels increase markedly in primary neurons, suggesting a protective or adaptive reprogramming response [33]. Beyond mRNA, m^1^A affects noncoding RNAs by modulating long noncoding RNA (lncRNA)-protein interactions and facilitating circular RNA (circRNA) translation, expanding its regulatory scope beyond protein-coding genes [34].

### 2.5. m^5^C: Developmental Regulation in the Brain

5-methylcytosine (m^5^C) is another RNA modification implicated in neurodevelopment and neural lineage specification [35]. Distinct m^5^C methylation patterns are observed between neural stem cells and differentiated neurons, suggesting a role for m^5^C in transcriptomic remodeling during neuronal differentiation and brain maturation [35].

Taken together, epitranscriptomic modifications such as m^6^A, m^1^A, and m^5^C are now recognized as critical regulators of mRNA fate in the nervous system. By dynamically modifying RNA, these marks provide a reversible, fine-tuned mechanism for adapting gene expression in response to developmental cues and environmental challenges. Continued investigation into RNA modifications will enhance our understanding of brain function and may reveal novel therapeutic targets for neurodevelopmental and neurodegenerative disorders.

### 2.6. Genetic and Environmental Influences on m^6^A and Disease Susceptibility

Mutations or dysregulation of the m^6^A methylation machinery can disrupt the processes of installing, removing, or interpreting the m^6^A mark [36,37]. Such alterations affect RNA decay and transcriptional output. In parallel, environmental factors, including psychosocial stress, nutrition, and exposure to xenobiotics, can act as stress-like stimuli that reshape the m^6^A landscape and reprogram cellular transcriptional responses [38,39]. When combined with heritable risk, these context-dependent influences render homeostatic m^6^A regulatory mechanisms vulnerable to destabilization and adaptive restructuring. The cumulative effects of genetic and environmental perturbations can lead to aberrant changes in neuroectoderm-derived lineages and their glial progeny, laying the groundwork for the emergence of polygenic neurodevelopmental and mood disorders [40,41].

## 3. m^6^A Methylation in Brain Development and Function

**Figure 1:**
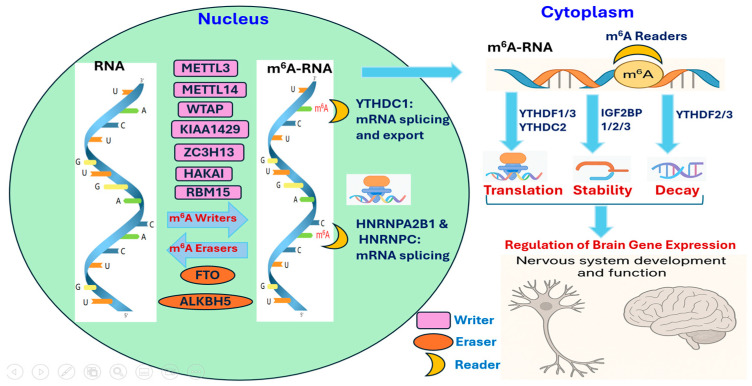
RNA Methylation Dynamics in the Nervous System.

m^6^A RNA methylation serves as a fundamental regulatory mechanism during brain development, modulating processes such as neurogenesis, dendritic and axonal development, and synaptic plasticity. As a dynamic and reversible epitranscriptomic mark, m^6^A influences the trafficking and local translation of mRNAs at synapses, a process essential for proper neuronal function [42]. m^6^A methylation is crucial for the timing and progression of neurogenesis. In the developing cortex, m^6^A facilitates the cell cycle progression of neural progenitor cells (NPCs), promoting their timely differentiation. Loss of m^6^A methyltransferase components such as METTL14 leads to prolonged NPC proliferation and delayed neuronal subtype specification, indicating that m^6^A promotes the decay of transcripts maintaining the progenitor state [43]. m^6^A marks are enriched on mRNAs encoding transcription factors and cell cycle regulators, highlighting their role in orchestrating neurodevelopmental gene expression programs [43]. In post-mitotic neurons, m^6^A regulates dendritic branching and axonal development. In retinal ganglion cells, the m^6^A reader protein YTHDF2 controls dendritic complexity by modulating the decay of target mRNAs. Knockdown of YTHDF2 increases dendritic branching, highlighting the role of m^6^A-mediated mRNA decay in shaping dendritic architecture [44]. Similarly, m^6^A modifications affect the subcellular localization and translation of mRNAs encoding axon guidance molecules and cytoskeletal components. Disruption of m^6^A pathways impairs axonal growth and pathfinding, underscoring the importance of m^6^A in establishing neural circuits [45]. m^6^A also plays a central role in synaptic regulation. Activity-dependent recruitment of the demethylase ALKBH5 to active synapses modulates local m^6^A levels, thereby regulating the translation of proteins involved in synaptic transmission and plasticity [46]. m^6^A-modified transcripts are enriched in dendrites and axons, enabling localized protein synthesis in response to neuronal activity, a process essential for synaptic remodeling, long-term potentiation (LTP), and memory formation. Figure 1 depicts RNA methylation dynamics and the roles of m^6^A regulators in neurodevelopment.

## 4. m^6^A Methylation and Learning and Memory

### 4.1. Mechanisms of m^6^A in Cognitive Function

Growing evidence supports m^6^A RNA methylation as a critical regulator of learning and memory. By modulating the stability, localization, and translation of brain mRNAs, m^6^A enables fine-tuning of synaptic gene expression in response to neuronal stimuli. This regulation affects transcripts encoding key synaptic proteins such as brain-derived neurotrophic factor (BDNF), cAMP response element-binding protein (CREB), and activity-regulated cytoskeleton-associated protein (Arc), all of which are essential for synaptic plasticity [47]. During neuronal activity, m^6^A levels are dynamically modulated at synaptic sites. Reader proteins such as YTHDF1 and YTHDF3 promote the local translation of m^6^A-modified mRNAs at activated glutamatergic synapses, facilitating long-term potentiation (LTP) and memory consolidation [46]. Genetic disruption of these reader proteins impairs learning and memory, indicating their essential role in cognitive processing. For instance, conditional knockout of METTL3 in the hippocampus leads to deficits in spatial learning and memory, while YTHDF1 deficiency disrupts memory retrieval [48]. Moreover, FTO, an m^6^A demethylase, exhibits dose-dependent effects on cognition, i.e., its overexpression enhances, and its depletion impairs, memory performance [49,50].

### 4.2. Studies on Memory Formation and Retrieval

Experimental studies in animal models further underscore the involvement of m^6^A in memory processes. In Drosophila, mutations in m^6^A writer and reader proteins impair short-term memory formation [51]. In mice, reader protein YTHDF1 promotes translation of memory-associated transcripts in the hippocampus, with its loss resulting in learning and memory deficits [48]. Depletion of FTO, an m^6^A demethylase, impairs object location memory in male mice, indicating that FTO-mediated demethylation plays a role in specific memory tasks [50]. In rats, knockdown of METTL3 in the hippocampus abrogated bisphenol A (BPA)-induced learning and memory deficit [52]. These findings confirm that both the installation and recognition of m^6^A marks are essential for proper memory formation, retrieval, and storage.

### 4.3. Therapeutic Implications for Memory Improvement

Targeting m^6^A pathways presents promising therapeutic avenues for memory-related disorders such as Alzheimer’s disease (AD) and age-associated cognitive decline. In 5 × FAD mouse model of AD, overexpression of METTL16 increases m^6^A levels, enhances synaptic plasticity, reduces Aβ_1–42_ accumulation, and improves cognitive performance [53]. Additionally, m^6^A-regulated genes such as SNRPG and SNRPD2 may serve as biomarkers for progression from mild cognitive impairment to AD [54]. Age-related declines in m^6^A methylation correlate with reduced synaptic protein synthesis and impaired cognition, suggesting that maintaining m^6^A homeostasis may be key to preserving brain function in aging populations [55]. Future therapeutic strategies may involve modulating m^6^A writer, eraser, or reader activity to restore optimal RNA methylation landscapes in the brain. Development of small molecules targeting these regulators could open novel pathways to prevent or treat cognitive dysfunction and neurodegenerative diseases.

## 5. m^6^A Methylation and Psychiatric Disorders

m^6^A RNA methylation plays a significant role in the regulation of mood and stress, with growing evidence linking it to psychiatric phenotypes. In depression models, the m^6^A demethylase FTO has been shown to influence hippocampal memory and stress adaptation [56]. FTO deficiency enhances fear memory consolidation and alters the expression of stress-responsive genes in the prefrontal cortex, suggesting that m^6^A dynamics are central to emotional regulation [56]. FTO also modulates neuroplasticity via the brain-derived neurotrophic factor (BDNF) pathway, a key player in mood regulation and synaptic function. In anxiety disorders, similar mechanisms appear to contribute to heightened stress sensitivity, as m^6^A patterns shift in response to environmental stressors. Moreover, genetic studies have linked schizophrenia risk to polymorphisms in *ZC3H13*, which is an m^6^A writer gene. RBM15, another component of the m^6^A “writer” complex, has been implicated in synapse formation and axonal growth, suggesting that disrupted m^6^A-mediated synaptic regulation may contribute to cognitive and behavioral impairments in psychiatric disorders [56]. Table 1 summarizes the associations between altered m^6^A RNA methylation or dysregulation of m^6^A regulatory genes and psychiatric disorders, with further details from these published studies provided below.

### 5.1. m^6^A Methylation and Depression

Compelling evidence suggests that m^6^A RNA methylation plays a critical role in the pathophysiology of major depressive disorder (MDD). The m^6^A demethylase FTO is highly expressed in the hippocampus, a brain region consistently implicated in depression. Li et al. demonstrated that reduced hippocampal FTO expression is associated with depression-like behaviors in rodents [66]. Similarly, human postmortem brain studies have shown that reduced FTO expression in the hippocampus [67], as well as reduced FTO expression and increased METTL3 expression in the dorsolateral prefrontal cortex (dlPFC) [68], are associated with MDD. Mechanistically, FTO deficiency disrupts β2-adrenergic receptor (ADRB2) signaling and impairs downstream CaMKII/CREB pathways, which are essential for synaptic plasticity and stress resilience. Moreover, FTO downregulation attenuates responses to common antidepressants, suggesting that m^6^A dynamics may influence treatment efficacy. Conversely, overexpression of FTO exerts antidepressant-like effects [58]. Further supporting the role of m^6^A in depression, Fan et al. demonstrated that dysregulation of m^6^A-related components, specifically the reader protein YTHDF1 and the writer METTL3, modulates neuroimmune signaling [59]. In particular, these alterations impact the TRAF6-NF-κB pathway, a key regulator of microglial activation and neuroinflammation, both of which are increasingly recognized as central to depressive symptomatology. Additionally, m^6^A modifications influence hippocampal neural stem cell proliferation and differentiation, thereby affecting neurogenesis and structural plasticity, both of which are frequently compromised in MDD. In depression, m^6^A modifications may also vary in their associations and effects across subtypes, for example, between treatment-resistant and typical depression, with stronger impacts observed on stress-response systems and signaling pathways [57,61]. Together, these findings underscore the multifaceted role of m^6^A methylation in shaping stress-response pathways, neuroplasticity, and neuroimmune interactions, and highlight its potential as a therapeutic target for depression.

### 5.2. m^6^A Methylation and Anxiety Disorders

Although m^6^A RNA methylation has been more extensively studied in depression, a growing body of evidence indicates its involvement in the molecular mechanisms underlying anxiety disorders. Epitranscriptomic regulation appears to influence gene expression within brain circuits critical for anxiety, particularly through the actions of m^6^A regulators such as FTO in limbic regions including the hippocampus, anterior cingulate cortex (ACC), and ventral tegmental area (VTA). In animal models, FTO deficiency in the VTA and hippocampus is associated with altered stress reactivity and impaired emotional regulation, implicating m^6^A methylation in anxiety-like behavior [45]. Notably, the behavioral consequences of FTO manipulation are region-specific. For instance, FTO knockdown in the ACC induces both depressive- and anxiety-like phenotypes, possibly via downregulation of brain-derived neurotrophic factor (BDNF) signaling. In contrast, FTO deletion in the dorsal and ventral hippocampus does not elicit significant anxiety phenotypes, highlighting the context-dependent nature of m^6^A dynamics in regulating emotional behavior.

Beyond its role in neural circuits, m^6^A methylation may also modulate anxiety through neuroimmune pathways. Key m^6^A regulators, such as METTL3 and FTO, have been shown to influence microglial and macrophage polarization via key inflammatory cascades, including the NF-κB and MAPK pathways [61]. Given the well-established role of neuroinflammation in the pathophysiology of anxiety, these findings suggest a mechanistic link between m^6^A methylation, neuroimmune signaling, and anxiety-like behaviors.

Emerging evidence also supports the role of m^6^A methylation in modulating neurotransmitter systems involved in anxiety, particularly the monoaminergic pathways. A recent preclinical study by Kanarik et al. demonstrated that pharmacological activation of the METTL3/METTL14 complex using CHMA1004, an m^6^A RNA methylation activator, produced significant anxiolytic-like effects in rats [60]. Behavioral tests such as the open field and elevated zero-maze revealed increased exploratory behavior and reduced stress-induced defecation, especially in males. Importantly, these anxiolytic effects occurred without signs of psychostimulant activity, as evidenced by unchanged 50-kHz ultrasonic vocalizations and stable dopamine release. Transcriptomic profiling following repeated CHMA1004 administration revealed differential expression of genes involved in dopaminergic neuron viability, neuroinflammatory signaling, and cellular stress responses, particularly in the striatum, frontal cortex, and VTA. These molecular changes were accompanied by region-specific alterations in catecholamine levels, suggesting that m^6^A methylation may exert its anxiolytic effects, at least in part, through epitranscriptomic regulation of monoamine neurotransmission. Thus, activation of the METTL3/METTL14 complex may represent a promising therapeutic target for novel anxiolytic interventions. In anxiety disorders, m^6^A modifications may affect signaling pathways and regulatory systems governing stress and neuroendocrine function, thereby influencing both vulnerability and symptom severity [69].

Taken together, these findings underscore the emerging importance of m^6^A methylation in shaping stress sensitivity and anxiety-like behaviors through coordinated modulation of neuronal circuits, immune signaling, and neurotransmitter systems.

### 5.3. m^6^A Methylation and Schizophrenia

Emerging evidence suggests that m^6^A RNA methylation plays a role in the pathophysiology of schizophrenia, particularly through its regulation of synaptic function and neurodevelopmental gene expression. A recent study by Angelin et al. investigated m^6^A methylation and other epigenetic markers in peripheral blood mononuclear cells from first-episode schizophrenia patients undergoing treatment with second-generation antipsychotics [62]. While global m^6^A levels did not differ significantly from healthy controls, treatment non-responders exhibited modestly elevated m^6^A methylation levels compared to responders [62]. This finding suggests a potential link between m^6^A dynamics and variability in antipsychotic treatment response. Fragile X mental retardation protein (FMRP), an RNA-binding protein, regulates cortical development, dendritic spine maturation, and synaptic plasticity. FMRP is a potential m^6^A reader protein, modulating neural differentiation through m^6^A-dependent mRNA nuclear export [63] and stabilizing m^6^A-marked mRNA targets [64]. Although indirect, dysregulation of FMRP and its interaction with m^6^A-modified transcripts may contribute to the cognitive and behavioral abnormalities observed in schizophrenia-spectrum disorders. Additionally, mutations associated with autism and schizophrenia can disrupt the m^6^A “reader” protein YTHDF1, resulting in impaired microtubule function and abnormal neurodevelopment [70]. Collectively, these findings support the hypothesis that m^6^A RNA methylation contributes to schizophrenia through post-transcriptional dysregulation of neurodevelopmental and synaptic gene networks.

### 5.4. m^6^A Methylation and Bipolar Disorder

Emerging evidence implicates m^6^A RNA methylation in the pathophysiology of mood disorders, including bipolar disorder. Transcriptomic profiling of the striatum has revealed differential expression of genes involved in dopaminergic neurotransmission, neuroplasticity, and stress response—processes frequently dysregulated in bipolar disorder. Notably, upregulated genes included Vwa5b2, previously associated with lithium responsiveness in bipolar patients [65], and Nxph3, a synaptic gene downregulated after loss of m^6^A in chronic stress models [69]. These findings suggest that enhancing m^6^A methylation via METTL3/METTL14 activation may modulate gene networks underlying affective instability and stress sensitivity. In bipolar disorder, m^6^A dysregulation may also affect genes associated with circadian rhythm and synaptic plasticity, potentially contributing to mood shifts and altered neural signaling [61,71]. Collectively, these results provide compelling preclinical evidence that m^6^A methylation contributes to mood regulation and treatment response in bipolar disorder, highlighting its potential as a target for novel epitranscriptomic therapies.

## 6. Future Directions and Limitations

Despite significant advances, numerous challenges remain in the study and clinical application of m^6^A methylation. Current epitranscriptomic methods lack sufficient resolution and sensitivity, likely missing many modification sites. In the brain, the complexity of m^6^A regulation is further compounded by its heterogeneous cellular architecture, which obscures cell-type-specific patterns and highlights the need for single-cell and spatially resolved analyses. Developing pharmacological agents that selectively target m^6^A methyltransferases, demethylases, or binding proteins without affecting other pathways remains particularly challenging. Because these regulators are broadly expressed and participate in multiple biological processes, global modulation risks adverse side effects. Moreover, existing technologies cannot yet engineer m^6^A marks in a transcript-specific, spatially localized, and cell-type-specific manner. Delivering m^6^A-targeted therapies to the brain poses another major hurdle, as both gene-based and small-molecule approaches require sophisticated delivery systems capable of crossing the blood–brain barrier without causing toxicity or triggering immune responses. Given the dynamic and reversible nature of m^6^A, longitudinal studies are needed to determine how transient versus chronic alterations affect brain function across development, aging, stress, and disease states. In addition, many current studies rely on rodent models or cultured cells, which may not fully capture the complexity of human psychiatric disorders. Integrating data from postmortem human brain tissues, patient-derived induced pluripotent stem cells (iPSC), and multi-omics strategies will be critical to establish the clinical relevance of m^6^A dysregulation. Importantly, m^6^A should be studied within the broader epigenetic landscape, in concert with DNA methylation, histone modifications, and noncoding RNAs, to fully understand its role in mental health and disease.

Another major limitation is the poorly defined relationship between DNA CpG methylation and RNA m^6^A modification. Whether CpG density influences m^6^A deposition or if they represent independent processes remains unresolved. Similarly, the mechanisms governing the deposition and removal of m^6^A in neurons are not well understood. While m^6^A is thought to be added co-transcriptionally and removed dynamically in both the nucleus and cytoplasm, the functional rationale for this regulation is still unclear. Interactions between m^6^A and other RNA processes, such as A-to-I editing, translational decoding, or ribosome kinetics, warrant further investigation. Likewise, the rules guiding site selection within DRACH motifs in neurons remain to be elucidated. Another open question concerns the impact of m^6^A on microRNA cleavage and target recognition. These unresolved issues point to fundamental gaps linking m^6^A to brain function and psychiatric disease. Addressing them will require functional studies integrated with epitranscriptomic and genomic analyses, ultimately clarifying how m^6^A contributes to vulnerability and resilience in mental illness.

## 7. Conclusions

This review highlights the expanding role of m^6^A RNA methylation in brain development, cognition, and neuropsychiatric disorders. By fine-tuning mRNA fate, m^6^A methylations shape neurogenesis, synaptic plasticity, and stress responses. Dysregulation of m^6^A pathways has been implicated in a range of conditions, including depression, anxiety, schizophrenia, bipolar disorder, and addiction. Clinically, m^6^A research opens new avenues for developing blood-based biomarkers and targeted therapeutics aimed at correcting specific molecular deficits. Modulating m^6^A turnover through selective inhibition or activation of methyltransferases, demethylases, or reader proteins holds promise for precision medicine, particularly in treatment-resistant cases. Looking ahead, major challenges remain, including achieving selective targeting, ensuring tissue- and cell-type-specific delivery, and establishing long-term safety. Comprehensive mapping of m^6^A methylation patterns across brain regions and developmental stages in humans will be vital for translating laboratory discoveries into effective clinical interventions. As technologies for probing and manipulating the epitranscriptome advance, m^6^A is poised to become a powerful lever for deepening our understanding of mental health and improving therapeutic outcomes.

## Figures and Tables

**Table 1:** The association of m^6^A RNA methylation/m^6^A regulators with psychiatric disorders.

Psychiatric Disorders	m^6^A Methylation/ m^6^A Regulators	Human/ Animal	Research Findings	References
Depression	FTO	Human	Polymorphisms linked to depression risk/protection	[56]
	FTO; ALKBH5	Human, mouse	Reduced hippocampal FTO leads to depression-like behaviors; Reduced VTA FTO leads to higher stress susceptibility; circSTAG1 overexpression inhibits ALKBH5 translocation, resulting in reduced depressive-like behaviors	[45]
	Global m^6^A	Human	Sex-specific m^6^A changes: microtubule movement in	[57]
		vmPFC	males; neuronal projection in females	
	FTO	Human	Decreased hippocampal FTO leads to MDD	[58]
	FTO	Mouse	Hippocampal Fto KD/KO leads to depression-like	[58]
			behaviors; Fto overexpression rescues; ADRB2 stimulation rescues	
	ALKBH5	Human	Decreased peripheral blood ALKBH5 leads to MDD	[58]
	Global m^6^A	Human & Animal	Altered brain m^6^A linked to depression	[59]
	METTL3; METTL14	Rat	Antidepressant-like effects; regulated depression/stress genes	[60]
Anxiety	FTO	Mouse	FTO deficiency reduced anxiety- and depression-like behaviors	[45]
disorders	METTL3	Mouse	Loss of METTL3 increases fear generalization	[45]
	FTO	Mouse VTA	FTO loss increases stress; overexpression protective	[45]
	FTO, global m^6^A	Mouse PFC	FTO knockdown increases cued fear memory	[45]
	FTO, ALKBH5, global m^6^A	Mouse	Stress alters m^6^A regionally	[61]
	FTO	Mouse	Reduced FTO in anterior cingulate cortex increases anxiety	[61]
	FTO (global KO)	Mouse	FTO (global KO) leads to anxiety	[61]
	METTL3; METTL14	Rat	METTL3/14 activation reduces anxiety-like behavior	[60]
Schizophrenia	ZC3H13	Human	Polymorphism associated with schizophrenia	[56]
	Global m^6^A	Human	m^6^A level unchanged; slightly up in non-responders (not significant)	[62]
	FMRP (m^6^A reader)	Mouse	FMRP loss leads to nuclear retention of m^6^A RNAs	[63]
	FMRP (m^6^A reader);	Mouse	FMRP stabilizes m^6^A-marked RNAs;	[64]
	YTHDF2		FMRP loss leads to YTHDF2-driven decay	
Bipolar	m^6^A	Human	DMR—EIF2B5/VWA5B2 (TTS) related to lithium response	[65]
disorder	m^6^A	Human	DMR—RALGAPA1 (promoter/TSS) related to lithium response	[65]
	m^6^A	Human	DMR—C2orf81 (exon) related to lithium response	[65]
	m^6^A	Human	DMR—LINC01237 (intron) related to lithium response	[65]
	m^6^A	Human	DMR—Intergenic sites related to lithium response	[65]
	METTL3/METTL14 activator compound CHMA1004	Rat	CHMA1004 elicits anxiolytic-like effects	[60]

vmPFC: ventral medial prefrontal cortex; VTA: ventral tegmental area; KD/KO: knockdown/knockout; MDD: major depressive disorder; DMR: differentially methylated region.

## Data Availability

This is a review article; no new data were created or analyzed, and therefore data sharing is not applicable.
